# Hair as a Means of Classifying Races

**Published:** 1883-06

**Authors:** E. B. Tylor


### HAIR AS A MEANS OF CLASSIFYING RACES.

A single hair now enables the anthropologist to judge in what division of the human species he will class its owner; there is no mistaking a Chinese for a European, or either for an African. The cross-section of this single hair, examined microscopically by Pruner's method, shows it circular, or oval, or reniform; its follicle curvature may be estimated by the average diameter of the curls as proposed by Moseley; its coloring matter may be estimated by Sorby's method. There has been even a systematic classification of man published by Dr. W. Muller, of the Novara Expedition, which is primarily arranged according to hair, in straight-haired race, curly-haired races, etc., with a secondary division according to language. Though we cannot regard such a system as good, the wonder is that it should answer so well as it does; indeed, nothing could prove more clearly how real race-distinctions are, that a single bodily character should form a basis for rationally mapping out the divisions of mankind.—*E. B. Tylor in Nature.*

---

IF the body is tired, rest; if the brain is tired, sleep.

IF the bowels are loose lie down; eat nothing until you are well.

NO SUBJECT is of more interest at the present day than that of the proper feeding and rearing of infants. It behooves every physician and every mother to investigate this important matter. There can be no question but that the breast milk of a healthy mother is the best food for a baby, for the sake of both the mother and the child.

When, however, human milk becomes so poor as to be no longer nutrituous, it ceases to rank as food; and by continuing to suckle a child with it, his stomach is filled with a fluid which is incapable of nourishing him, but which by satisfying his appetite for the moment prevents his taking a meal which would be really beneficial.

In such cases the best substitute should be sought. Until Liebig made known his formula, a real and perfect substitute for mother's milk was unknown.

Mellin's food is made from Liebig's formula, with such modification as renders it fit for commercial purposes. It is in the form of a dry extract or powder, contains nothing farinaceous, and is entirely soluble. Its mode of preparation is very simple, requiring only to be dissolved in hot milk and water, and when so dissolved it forms the most perfect substitute for mother's milk, that has yet been obtained. The proprietors, Messrs. Metcalf & Co.,of 41 Central Wharf, Boston, have prepared a very useful pamphlet, fully explaining the food, which they will send free to any one desiring it. It contains much useful information, and should be in the hands of every mother.

